# Leukotriene B4 activates intracellular calcium and augments human osteoclastogenesis

**DOI:** 10.1186/s13075-014-0496-y

**Published:** 2014-12-02

**Authors:** Neha Dixit, Dennis J Wu, Yesser H Belgacem, Laura N Borodinsky, M Eric Gershwin, Iannis E Adamopoulos

**Affiliations:** Division of Rheumatology, Allergy and Clinical Immunology, University of California, Davis, 451 Health Sciences Drive, CA 95616 USA; Department of Physiology and Membrane Biology, Shriners Hospitals for Children–Northern California, Sacramento, 2425 Stockton Blvd, CA 95817 USA; Institute for Pediatric Regenerative Medicine, Shriners Hospitals for Children–Northern California, Sacramento, 2425 Stockton Blvd, CA 95817 USA

## Abstract

**Introduction:**

Bone erosion in inflammatory arthritis depends on the recruitment and activation of bone resorbing cells, the osteoclasts. Interleukin-23 (IL-23) has been primarily implicated in mediating inflammatory bone loss via the differentiation of Th17 receptor activator of nuclear factor κB ligand (RANKL)–producing cells. In this article, we describe a new role of IL-23 in activating the synthesis and production of leukotriene B4 (LTB4) in innate immune cells.

**Methods:**

We utilized whole blood–derived human peripheral blood mononuclear cells (PBMCs), differentiated them towards an osteoclast lineage and then performed immunofluorescence and cytochemical staining to detect the expression of LTB4-associated receptors and enzymes such as phospholipase A2, 5-lipoxygenase and leukotriene A4 hydrolase, as well as the presence of tartrate-resistant acid phosphatase (TRAP) and F-actin rings on fully mature osteoclasts. We used enzyme immunoassays to measure LTB4 levels in culture media derived from IL-23-treated human PBMCs. We used real-time calcium imaging to study the effect of leukotrienes and requirements of different calcium sources and signaling proteins in activating intracellular calcium flux using pharmacological inhibitors to phospholipase C (U73122), membrane calcium channels (2-APB) and phosphatidylinositol 3-kinase (Wortmannin) and utilized qPCR for gene expression analysis in macrophages and osteoclasts.

**Results:**

Our data show that LTB4 engagement of BLT1 and BLT2 receptors on osteoclast precursors leads to activation of phospholipase C and calcium release–activated channel–mediated intracellular calcium flux, which can activate further LTB4 autocrine production. IL-23-induced synthesis and secretion of LTB4 resulted in the upregulation of osteoclast-related genes *NFATC1, MMP9, ACP5, CTSK* and *ITGB3* and the formation of giant, multinucleated TRAP^+^ cells capable of F-actin ring formation. These effects were dependent on Ca^2+^ signaling and were completely inhibited by BLT1/BLT2 and/or PLC and CRAC inhibitors.

**Conclusions:**

In conclusion, IL-23 can initiate osteoclast differentiation independently from the RANK-RANKL pathway by utilizing Ca^2+^ signaling and the LTB4 signaling cascade.

## Introduction

In inflammatory arthritis, pathological bone erosion occurs because of increased differentiation and activation of osteoclasts, the only specialized bone-resorbing cells. Under physiological conditions, osteoclasts are derived from c-fms^+^/RANK^+^ monocyte/macrophage precursor cells and develop into fully functional osteoclasts upon receptor engagement by their ligands macrophage colony-stimulating factor (M-CSF) and receptor activator of nuclear factor κB ligand (RANKL) [1]. Once terminally differentiated, these osteoclasts adhere to the bone surface via α_v_β_3_ integrins, reorganize their cytoskeleton to form actin-rich sealing zones and secrete enzymes such as tartrate-resistant acid phosphatase (TRAP), cathepsin K and matrix metalloproteinase 9 (MMP9), which facilitate bone resorption [2]. Whereas RANKL signaling determines osteoclastogenesis under physiological conditions, several proinflammatory cytokines, including interleukin 23 (IL-23), IL-17 and tumor necrosis factor (TNF) can also activate osteoclastogenesis and exacerbate inflammation in the joint tissue [3-5]. Hence, it is crucial to study these alternate pathways and their role in mediating inflammatory arthritis.

IL-23 has been implicated primarily in mediating inflammatory bone loss via the differentiation of Th17 cells and the production of pro-osteoclastogenic cytokines IL-17, RANKL and TNF [6]. We recently demonstrated that IL-23 gene transfer in mice rapidly induced synovial inflammation and osteoclastogenesis in the absence of T cells [5]. G protein–coupled receptors (GPCRs) possess the ability to transmit intracellular signals within milliseconds of activation, whereas growth factor and cytokine receptors lack this rapidity and specificity in signaling [7,8]. Thus, this rapid induction of inflammation observed during IL-23 gene transfer prompted us to investigate, alternate inflammatory pathways associated with GPCRs. One pathway that has been associated with rapid inflammation and osteoclast formation is the leukotriene activation pathway [9].

Leukotrienes are active lipid mediators of inflammation generated primarily from myeloid leukocytes such as neutrophils, monocytes, macrophages and mast cells from the metabolism of arachidonic acid via the 5-lipoxygenase (5-LO) pathway [10]. This arachidonic acid is first generated from phospholipids via the activity of the calcium-dependent cytosolic phospholipase A_2_ (PLA_2_) [11], which provides the initial step in the leukotriene biosynthesis cascade. Leukotrienes consist of leukotriene B4 (LTB4) and the cysteinyl leukotrienes: namely, leukotriene C4 (LTC4), leukotriene D4 (LTD4) and leukotriene E4 (LTE4). These are all produced from leukotriene A4 (LTA4) by the differential activity of either LTA4 hydrolase (LTA4H) or LTC4 synthase (LTC4S) [12]. BLT1 and BLT2 are high- and low-affinity GPCRs, respectively, for LTB4 [13,14], and studies using BLT1-deficient mice have demonstrated a resistance to inflammatory arthritis and significantly reduced bone destruction [9,15]. A similar phenotype is observed in mouse strains deficient in LTB4 biosynthesis enzymes such as 5-LO and LTA4H, which collectively highlight the significance of LTB4 in inflammatory arthritis and osteoclastogenesis [16,17]. In keeping with these observations, LTB4 levels have also found to be elevated in the synovial fluid and tissue of patients with rheumatoid arthritis and are associated with several other inflammatory disorders, including psoriasis and bronchial asthma [18,19].

In this study, we investigated the dynamics between IL-23 and LTB4, two inflammatory mediators that may orchestrate osteoclast differentiation and activation in inflammatory arthritis. We previously demonstrated that systemic IL-23 expands the CD11b^+^Gr1^high^ myeloid subpopulation, which comprises the primary cell type involved in the biosynthesis of LTB4 [5,17]. In this study, for greater clinical significance, we demonstrate that treatment of human peripheral blood mononuclear cells (PBMCs) with IL-23 activates the release of LTB4. This LTB4 can engage with its receptors BLT1 and BLT2, which are receptors on macrophages leading to activation of phospholipase C (PLC) and calcium release–activated channel (CRAC)–mediated intracellular calcium flux. LTB4 can also activate nuclear factor of activated T-cells, cytoplasmic 1 (NFATC1), and transcription of downstream osteoclast-related genes such as TRAP, cathepsin K and β_3_ integrin, as well as the formation of giant multinucleated TRAP^+^ cells with F-actin ring structures independent of RANKL. IL-23 can initiate osteoclast differentiation independently from the RANK-RANKL pathway, and it may utilize the LTB4 signaling cascade to drive the precursor cells toward osteoclast development. Blockade of the LTB4 pathway is therefore a potential therapeutic target for inflammatory arthritic diseases.

## Methods

### Antibodies and reagents

Human PBMCs were isolated from whole-blood filters from healthy donors obtained from Delta Blood Bank (Stockton, CA, USA). All protocols were approved by the University of California at Davis Institutional Review Board, and written informed consent was obtained as required. All cell incubations were performed in culture medium consisting of α minimal essential medium (Invitrogen, Carlsbad, CA, USA), 2 mM glutamine, 10% heat-inactivated fetal bovine serum (Invitrogen), 100 IU/ml penicillin and 100 μg/ml streptomycin. Human M-CSF, RANKL, IL-23 were purchased from R&D Systems (Minneapolis, MN, USA). Antibodies to 5-LO, LTA4H (EPR5713), BLT1 (202/7B1) and BLT2 were obtained from Abcam (Cambridge, UK), AbD Serotec (Raleigh, NC, USA) and Sigma-Aldrich (St Louis, MO, USA), respectively. Alexa Fluor 555 goat anti-rabbit secondary antibody was purchased from Life Technologies (Carlsbad, CA, USA) and fluorescein goat anti-mouse secondary antibody was purchased from Invitrogen. Fluo-4 AM (calcium dye) was purchased from Invitrogen. PLC inhibitor U73122 was purchased from Cayman Chemical (Ann Arbor, MI, USA). 2-Aminoethoxydiphenyl borate (2-APB) was purchased from Sigma-Aldrich. Phospho-PLA_2_ antibody (S505) was purchased from Abcam. LTB4, BLT1 and BLT2 antagonists (U-75302 and LY255283) and Wortmannin were purchased from Cayman Chemical. LTB4 was detected using an LTB4 enzyme-linked immunoassay (EIA) kit (Cayman Chemical) according to the manufacturer’s instruction.

### Osteoclast differentiation from human peripheral blood mononuclear cells

Human PBMCs were isolated by gradient density centrifugation using Histopaque-1077 cell separation medium (Sigma-Aldrich) as previously described [20]. Briefly, 1 × 10^5^ human cells were plated on 96-well plates on glass coverslips cultured for 24 hours in the presence of M-CSF (25 ng/ml), and then adhered cells were transferred to 24-well plates where they were cultured with either M-CSF (25 ng/ml), M-CSF (25 ng/ml) + RANKL (30 ng/ml) or M-CSF (25 ng/ml) + LTB4 (10 nM) for up to 14 days. Multinucleated (three or more nuclei), TRAP^+^ cells capable of F-actin ring formation, were characterized as osteoclasts. The cells cultured on plastic dishes were stained for TRAP using a commercially available kit (Sigma-Aldrich) according to the manufacturer’s instructions. F-actin ring formation was visualized using phalloidin-fluorescein isothiocyanate (FITC) staining (Sigma-Aldrich). Culture medium was collected and frozen at −80°C until EIA analysis.

### Immunofluorescence staining

Human PBMCs were isolated and cultured for 8 or 14 days with human M-CSF (25 ng/ml) or M-CSF (25 ng/ml) + RANKL (30 ng/ml). At the time of harvest, cells were fixed with 4% paraformaldehyde (PFA) at room temperature (RT) for 30 minutes, permeabilized with 0.5% Triton X-100 for 5 minutes, washed with phosphate-buffered saline (PBS) and then blocked with 50% goat serum for 20 minutes. Cells were then incubated with primary antibodies against LTB4 biosynthetic pathway proteins, including 5-LO, LTA4H, LTB4 receptors BLT1 and BLT2, and p-PLA_2_ at 4°C overnight, followed by incubation with fluorescent secondary antibody at RT for 1 hour, and then the cells were washed three times with PBS and mounted with mounting medium containing 4′,6-diamidino-2-phenylindole. For LTB4 receptor and phosphatidylinositol 3-kinase (PI3K) inhibition experiments, cells were pretreated with either ethanol control, both BLT1 U-75302 (100 nM) [21] and BLT2 antagonists LY255283 (100 nM) [22], or Wortmannin (1 μM) [23] for 15 minutes at 37°C before acute activation with media, 10 nM LTB4 or 100 ng/ml IL-23 and PFA fixation. Appropriate isotype control antibodies were used as required.

### Real-time calcium measurements

Human PBMCs were cultured with human M-CSF (25 ng/ml) for 8 days. On the eighth day, cells were replenished with PBS + 1.5 mM calcium just prior to the experiments. Cells were labeled with 3 μM fluo-4 AM, and 100 μl of media, 10 nM LTB4 or 100 ng/ml IL-23 was added acutely during calcium imaging. For LTB4 receptor, PLC, CRAC and PI3K inhibition experiments, cells were treated with either ethanol control, both BLT1 U-75302 (100 nM) and BLT2 antagonists LY255283 (100 nM), PLC inhibitor U73122 (1 μM) [24], 2-APB (100 μM) [25] or Wortmannin (1 μM) for 15 minutes at 37°C before calcium measurements. Fluo-4 AM intensity was measured and tracked over time using NIS-Elements BR software (Nikon Instruments, Melville, NY, USA).

### Real-time PCR

Human PBMCs were treated with M-CSF (25 ng/ml) or M-CSF (25 ng/ml) + LTB4 (10 nM) for 8 or 14 days, respectively. mRNA was isolated using an RNeasy Mini Kit (QIAGEN, Carpinteria, CA, USA), and cDNA was synthesized using the Omniscript Reverse Transcription Kit (QIAGEN). Message expression levels of *NFATC1, MMP9, ACP5 (TRAP), CTSK (cathepsin K)* and *ITGB3 (β*_*3*_*integrin)* were assessed using a SYBR Green–based quantitative real-time PCR system. Gene expression was calculated using the comparative cycle threshold (2^−ΔΔCt^) method (using the mean cycle threshold (Ct) value for 18S rRNA and the gene of interest for each sample). The equation 1.8*e* (Ct 18S rRNA − Ct gene of interest) × 10^4^ was used to obtain the normalized values.

### Statistical analysis

Data were analyzed by Student’s *t*-test. The significance values were set as follows: *P <0.05, **P < 0.01 and ***P < 0.001. All data are representative of at least three experiments, unless otherwise indicated.

## Results

### LTB4 biosynthetic enzymes and receptors are expressed in both macrophages and osteoclasts *in vitro*

**Figure 1:**
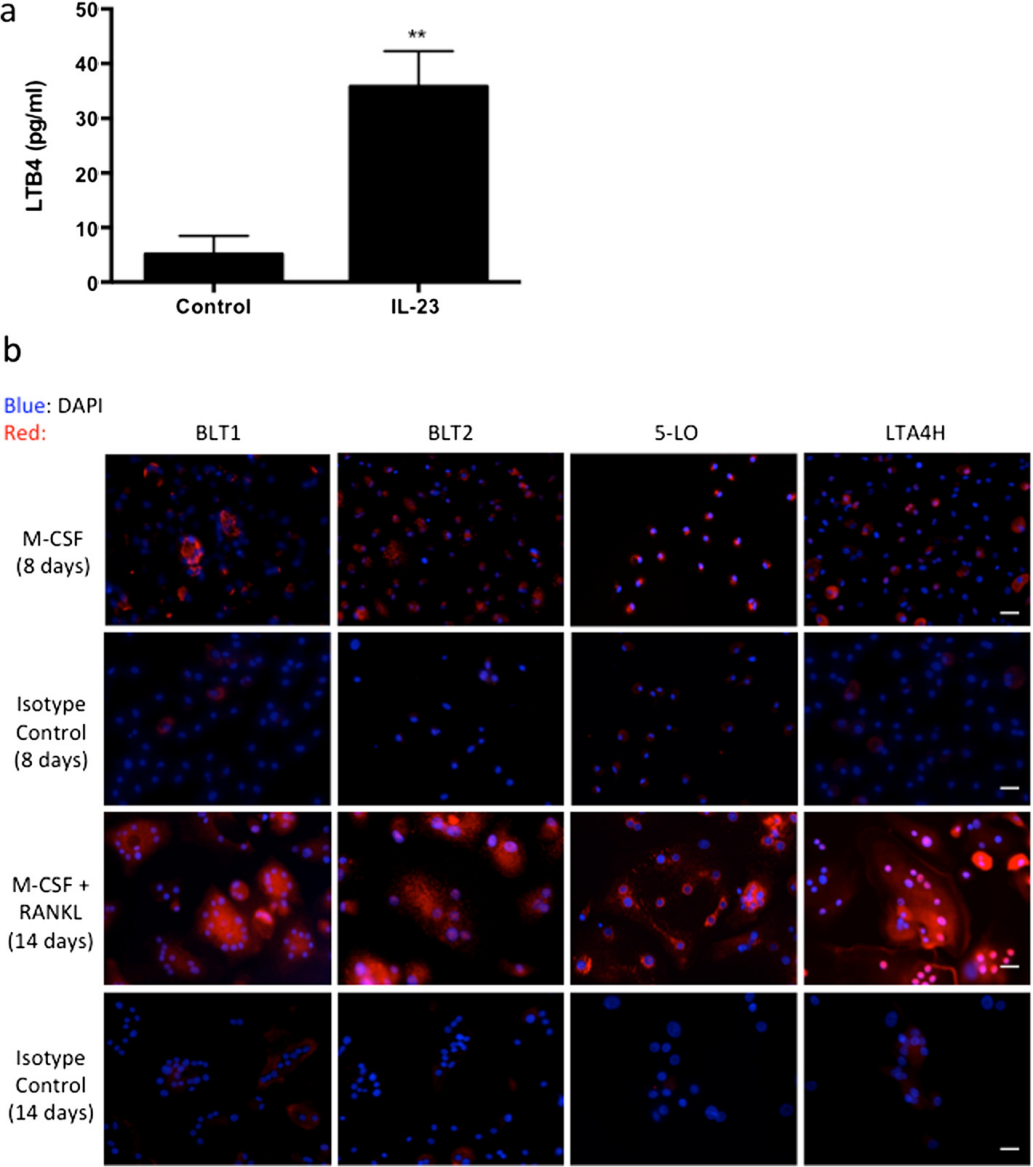
**Leukotriene B4 biosynthetic enzymes and receptors are expressed in both macrophages and osteoclasts. (a)** Quantification of leukotriene B4 (LTB4) levels by enzyme-linked immunoassay in the conditioned medium of human peripheral blood mononuclear cells (PBMCs) treated with 25 ng/ml macrophage colony-stimulating factor (M-CSF) and 10 ng/ml interleukin 23 (IL-23) for 3 days. Representative data from three experiments are shown. **P < 0.01. **(b)** Immunofluorescence of human PBMCs cultured with either M-CSF alone for 8 days or M-CSF + receptor activator of nuclear factor κB ligand (RANKL) for 14 days showing the expression of BLT1, BLT2, 5-lipoxygenase (5-LO) and leukotriene A4 hydrolase (LTA4H) in mononuclear and multinucleated giant cells. Representative images from three experiments are shown. 4′,6-diamidino-2-phenylindole (DAPI) is blue and BLT1, BLT2, 5-LO and LTA4H are in red. The scale bars represent 20 μm in Figure 1b.

IL-23 has been implicated primarily as a mediator of inflammatory bone loss via the activation of IL-17 production [26]. However, IL-23 may also activate innate immune cells to produce inflammatory mediators, such as leukotrienes, to amplify these inflammatory signals. We first demonstrated that exogenous addition of IL-23 in *in vitro* human PBMCs cultured in the presence of M-CSF for 3 days was sufficient to elevate the levels of LTB4 in the conditioned medium as compared to control cultures treated with M-CSF alone (M-CSF + IL-23: 35.79 ± 3.7 pg/ml, M-CSF: 5.1 ± 2.0 pg/ml, *P* < 0.01) as detected by EIA (Figure 1a). To investigate whether the expression of LTB4 is associated with cells of myeloid origin, human PBMC adherent cells (devoid of nonadherent lymphocytes) were cultured for 8 days in the presence of M-CSF. We determined by immunofluorescence that the biosynthetic enzymes involved in LTB4 production, 5-LO and LTA4H as well as LTB4 receptors BLT1 and BLT2, were present on macrophages (Figure 1b). To investigate whether this expression is maintained in terminally differentiated multinucleated osteoclasts, PBMC adherent cells were cultured for 8 days in the presence of M-CSF and further differentiated by exogenous addition of RANKL for 6 days. As in macrophages, multinucleated giant cells also expressed both the biosynthetic enzymes involved in LTB4 production (5-LO and LTA4H) and LTB4 receptors BLT1 and BLT2 (Figure 1b). Collectively, our data show that macrophages and osteoclasts can both express and respond to LTB4.

### LTB4 activates intracellular calcium flux via BLT1 and BLT2 receptors and via a PLC- and CRAC-dependent pathway

**Figure 2:**
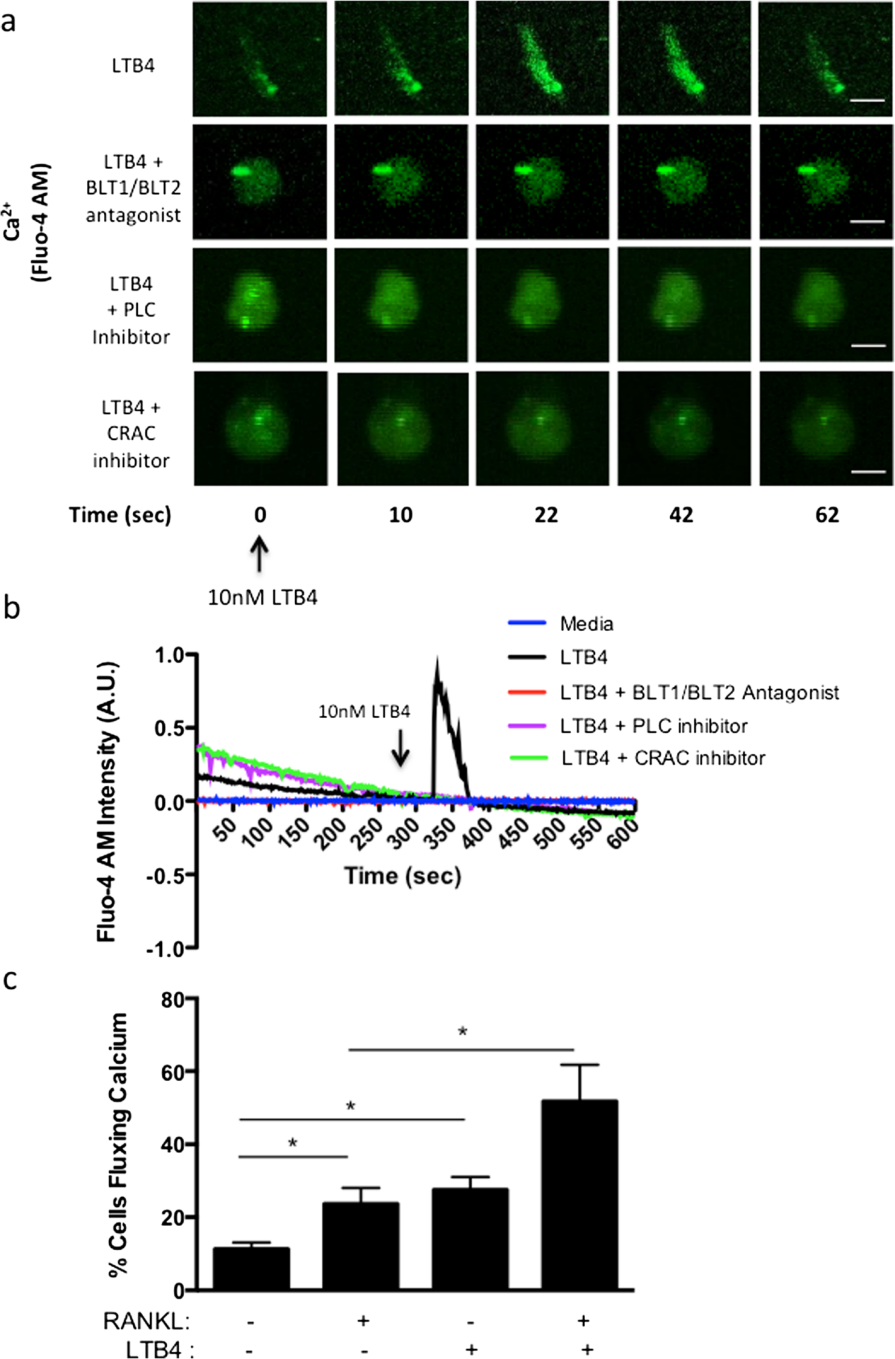
**Leukotriene B4 activates intracellular calcium flux. (a)** Intracellular calcium flux measurements in human peripheral blood mononuclear cells (PBMCs) cultured with macrophage colony-stimulating factor (M-CSF) for 8 days, labeled with fluo-4 AM, pretreated with either dimethyl sulfoxide control, 100 nM BLT1 and BLT2 antagonist cocktail, 1 μM phospholipase C (PLC) inhibitor U73122 or 100 μM calcium release–activated channel (CRAC) inhibitor 2-aminoethoxydiphenyl borate for 15 minutes prior to imaging in real time followed by acute activation with 10 nM LTB4. Representative data from three experiments are shown. The scale bars represent 10 μm. **(b)** Normalized fluo-4 AM intensity plotted for representative cells over the 600-second time course. **(c)** Graphical representation of the percentage of cells fluxing calcium in human PBMCs cultured with M-CSF for 8 days, labeled with fluo-4 AM and acutely activated with either 100 ng/ml receptor activator of nuclear factor κB ligand (RANKL), 10 nM LTB4 or both simultaneously. Representative data and images from three experiments are shown. *P < 0.05.

Because macrophages and osteoclasts express LTB4 receptors, we next sought to investigate the effects of LTB4 in macrophage-to-osteoclast differentiation. We observed that exogenous addition of LTB4 in *in vitro* human PBMCs cultured in the presence of M-CSF for 8 days was sufficient to induce calcium flux peaks 20 seconds after stimulation as compared to addition of media alone (LTB4 addition: 27.5 ± 2.5% cells, media addition: 11.25 ± 1.5% cells, *P* < 0.05) (Figure 2a, b and c). The observed calcium flux was completely abrogated in cell cultures pretreated with 100 nM of BLT1 and BLT2 antagonists (Figure 2a and b). Because these receptors are G protein–coupled and, once bound to LTB4, release PLC-dependent endoplasmic reticulum stores of calcium, we treated the cells with a PLC inhibitor to further understand the sequence of events. As expected, 8-day M-CSF differentiated PBMC cultures pretreated with the PLC inhibitor U73122 (1 μM) also lacked any calcium flux response (Figure 2a and b). Because the interplay between internal calcium stores and external calcium channels plays a significant role in maintaining calcium homeostasis within a cell, we also studied the requirement of membrane CRAC in LTB4-induced calcium flux [27,28]. Selective inhibition of CRAC channels with the small molecule inhibitor 2-APB (100 μM) completely abrogated the calcium response (Figure 2a and b). We ensured sufficient presence of extracellular calcium with the addition of 1.5 mM calcium to the cell cultures prior to measurements. Moreover, pretreatment with the above-mentioned BLT1/BLT2 antagonists, PLC and CRAC inhibitors did not decrease the viability of the cells as compared to the untreated cells, suggesting a key PLC- and CRAC–driven, calcium-dependent pathway following LTB4 stimulation (data not shown).

Intracellular calcium levels play a critical role in osteoclastogenesis. Therefore, we compared the calcium flux elicited by LTB4 in our cultures with RANKL, a potent osteoclastogenic factor. Our data show no significant difference in the percentage of cells responding with calcium activity between LTB4 (27.5 ± 2.5% cells) and RANKL (23.67 ± 2.5% cells) (Figure 2c). However, simultaneous activation with both RANKL and LTB4 induced 51.17 ± 5.2% cells to flux calcium, which was significantly higher than the treatments with RANKL or LTB4 alone (*P* < 0.05 with RANKL and *P* = 0.053 with LTB4). This suggests that RANKL and LTB4 can act synergistically to effectively signal for osteoclast differentiation.

### IL-23 induces phosphorylation of PLA_2_ in macrophages via PI3K

**Figure 3:**
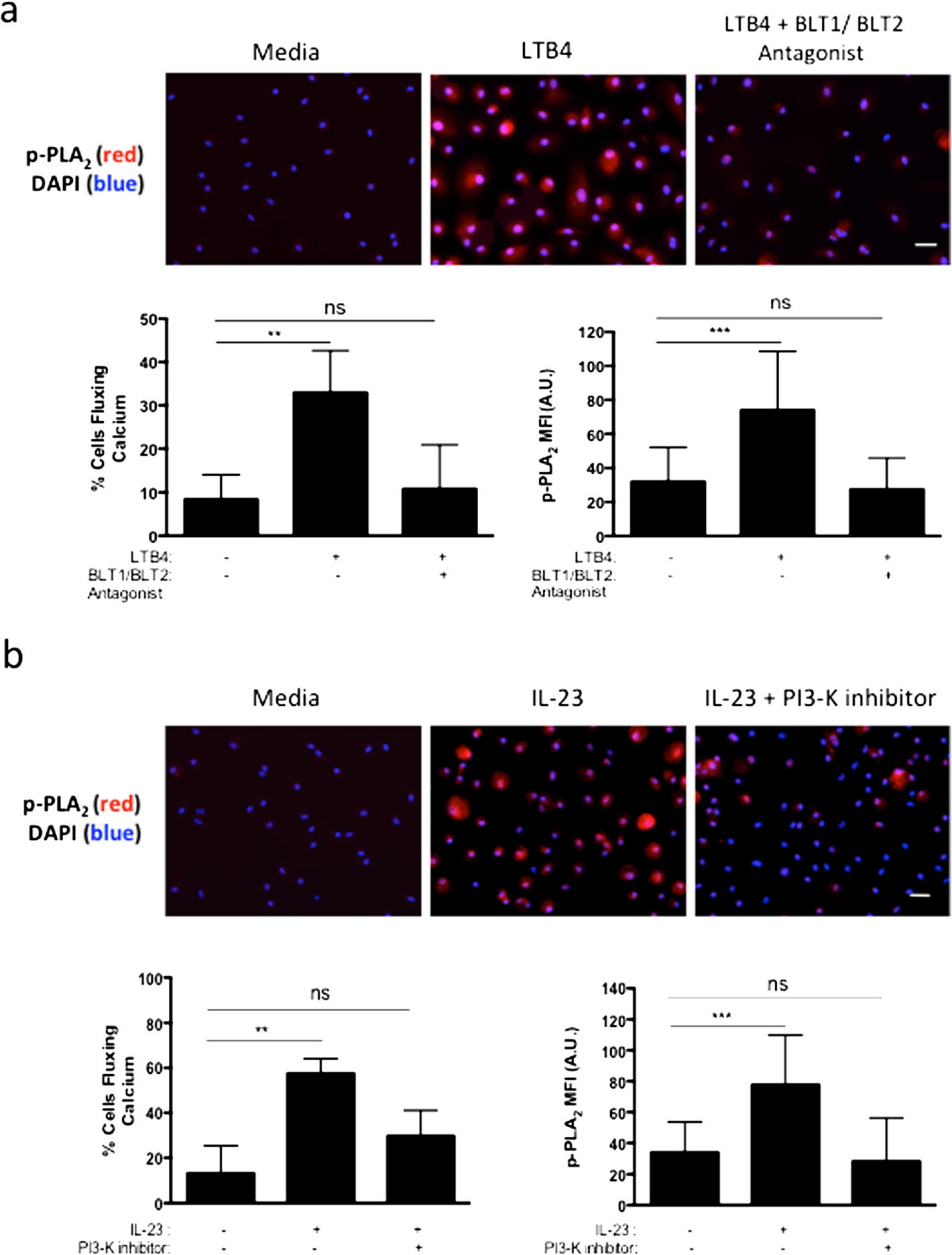
**Human interleukin 23 activates the leukotriene B4 synthesis pathway in macrophages via phosphatidylinositol 3-kinase. (a)** Graphical representation of the percentage of cells fluxing calcium and immunofluorescence imaging of phosphorylated phospholipase A_2_ (p-PLA_2_) in human peripheral blood mononuclear cells (PBMCs) cultured with macrophage colony-stimulating factor (M-CSF) for 8 days, followed by acute treatment of 10 nM leukotriene B4 (LTB4) with or without pretreatment of 100 nM BLT1 and BLT2 antagonists. **(b)** Graphical representation of the percentage of cells fluxing calcium and immunofluorescence imaging of p-PLA_2_ in human PBMCs cultured with M-CSF for 8 days, acutely activated with 100 ng/ml of interleukin 23 (IL-23) with or without pretreatment of 1 μM Wortmannin for 30 minutes. For calcium experiments, cells were also prelabeled with fluo-4 AM calcium dye. For p-PLA_2_ expression, cells were fixed, permeabilized and labeled for p-PLA_2_, and mean fluorescence intensity (MFI) was measured for >30 cells. PI3-K, Phosphatidylinositol 3-kinase. 4′,6-diamidino-2-phenylindole (DAPI) is blue and p-PLA_2_ expression is in red (scale bars represent 20 μm). Representative data and images from three experiments are shown. **P < 0.01 and ***P < 0.001.

Intracellular calcium flux, which induces osteoclastogenesis, also plays a role in activating the leukotriene biosynthesis pathway via the PLA_2_ pathway. Using a phospho-PLA_2_ antibody, we detected by immunofluorescence in 8-day M-CSF differentiated PBMC cultures, an induction of PLA_2_ phosphorylation after treatment with 10 nM LTB4 acutely for 10 minutes as compared with treatment with the media control (*P* < 0.001) (Figure 3a). PLA_2_ phosphorylation was commensurate with a threefold increase in the percentage of cells fluxing calcium over the media-alone control (LTB4: 32.9 ± 5.6% cells, media: 8.4 ± 2.9% cells, *P* < 0.01). The baseline of PLA_2_ phosphorylation in the controls is attributable to the presence of low levels of serum in the media, which were necessary for the calcium experiments and can activate mitogen-activated protein kinases (MAPKs) that phosphorylate PLA_2_ [11]. The LTB4-induced phosphorylation was restored to baseline levels in the presence of 100 nM BLT1 and BLT2 antagonists. Thus, our results clearly demonstrate that LTB4 can act via its receptors BLT1 and BLT2 to significantly activate the phosphorylated form of PLA_2_, which is an important mediator of downstream LTB4 production.

Next, we investigated whether IL-23 could activate the LTB4 synthesis pathway directly in macrophages. PI3K associates with the IL-23 receptor SH2 docking site at Tyr397 and can also phosphorylate PLA_2_ at Ser505 [29,30]. Hence, we investigated the requirement of PI3K in IL-23-mediated LTB4 production. Because phosphorylation of PLA_2_ is dependent on intracellular calcium levels, we first measured IL-23-mediated calcium flux in 8-day M-CSF treated PBMCs. Acute addition of 100 ng/ml IL-23 induced a threefold increase in the percentage of cells fluxing calcium as compared to the addition of media alone (IL-23: 57.5 ± 3.8% cells, media: 13.3 ± 5.4% cells, *P* < 0.01) (Figure 3b). Pretreatment with 1 μM Wortmannin, a potent PI3K inhibitor, for 15 minutes reduced the ability of cells to flux calcium via IL-23 stimulation by almost 50%. Furthermore, treatment of these macrophages with 100 ng/ml IL-23 for 10 minutes also elevated the expression of the phosphorylated form of PLA_2_ by twofold over media-only controls as measured by immunofluorescence, and this increased expression was interrupted to baseline levels in the presence of Wortmannin (Figure 3b). Notably, treatment with Wortmannin did not affect the cell viability as compared to the untreated cells (data not shown).

### LTB4 initiates osteoclastogenesis independent of RANKL signaling

**Figure 4:**
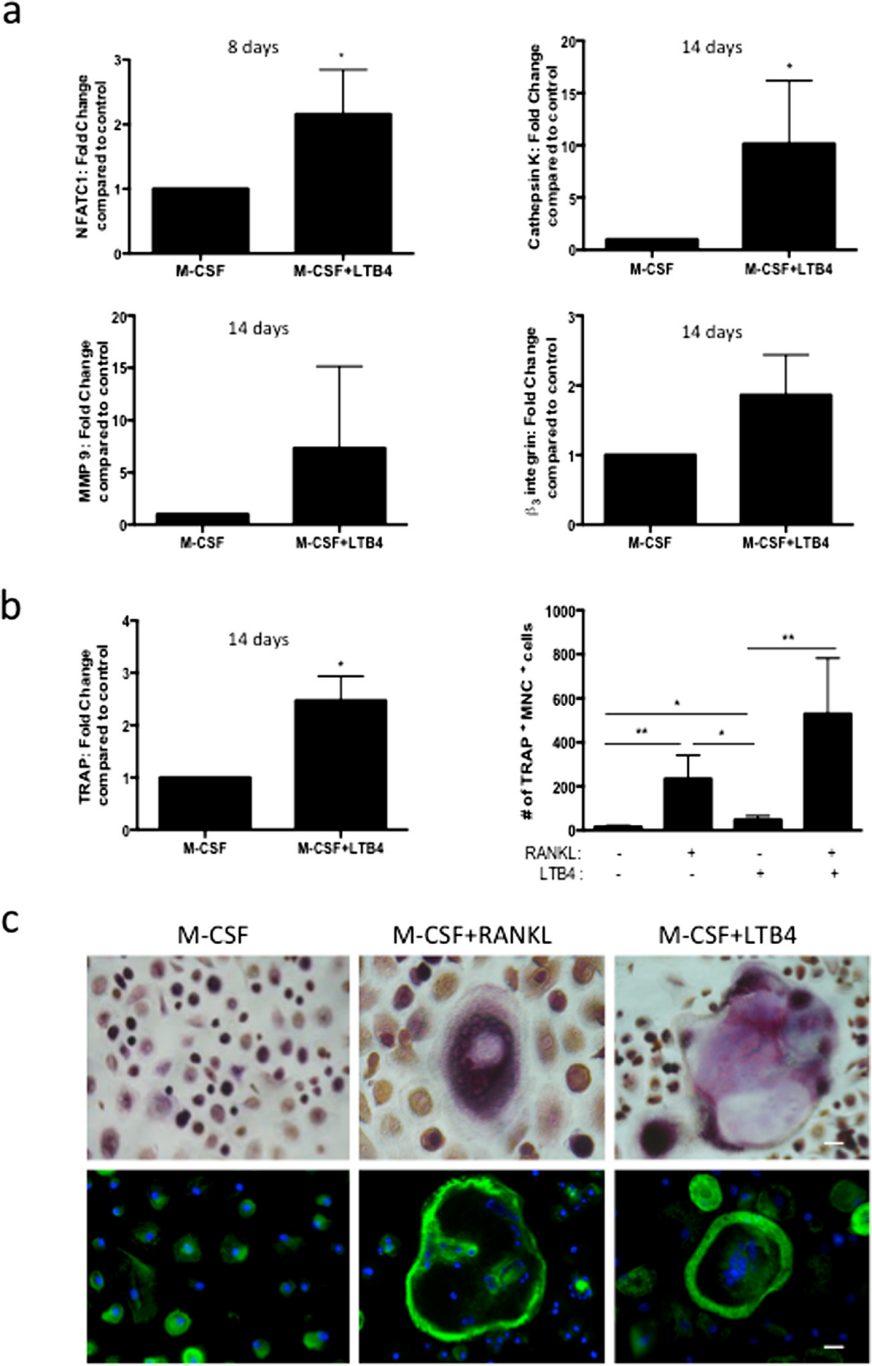
**Leukotriene B4 initiates osteoclastogenesis independent of receptor activator of nuclear factor κB ligand signaling. (a)** and **(b)** Gene expression analysis of human peripheral blood mononuclear cell (PBMCs) cultured with macrophage colony-stimulating factor (M-CSF), or M-CSF + leukotriene B4 (LTB4), for 8 or 14 days, showing the differential expression of nuclear factor of activated T-cells, cytoplasmic 1 (NFATC1), cathepsin K, tartrate-resistant acid phosphatase (TRAP), matrix metalloproteinase 9 (MMP9) and β_3_ integrin. **(b)** Cytochemical staining for TRAP in human PBMCs cultured with M-CSF, M-CSF + RANKL, M-CSF + LTB4, and M-CSF + RANKL + LTB4 for 14 days, showing the number of TRAP^+^ multinuclear cells (three or more nuclei) per frame of view. **(c)** Imaging of TRAP cytochemical staining and phalloidin staining in PBMCs cultured with M-CSF, M-CSF + RANKL, or M-CSF + LTB4 for 14 days (scale bars represent 20 μm). Representative data and images from three experiments are shown. *P < 0.05 and **P < 0.01.

Because LTB4 can activate calcium signaling independently of the canonical RANKL-RANK pathway in macrophages, we were interested in further investigating whether LTB4 was able to induce osteoclastogenesis independently of RANKL. We first assessed whether LTB4 stimulation was able to activate the transcription of osteoclast-related genes. Human PBMCs stimulated for 14 days with M-CSF + LTB4 displayed markedly increased expression in message levels of *NFATC1* (2-fold, *P* < 0.05), *cathepsin K* (10-fold, *P* < 0.05), *MMP9* (7-fold, *P* = 0.2024), *TRAP* (2.5-fold, *P* < 0.05) and *β*_*3*_*integrin* (2-fold, *P* = 0.056) as compared to treatment with M-CSF alone (Figure 4a and b). We further isolated human PBMCs and treated them with M-CSF, or M-CSF + RANKL, or M-CSF + LTB4, over a period of 14 days and measured osteoclastogenesis by quantitative analysis of TRAP^+^ multinucleated cells capable of F-actin ring formation (Figure 4b and c). Interestingly, LTB4 was able to form giant multinuclear TRAP^+^ cells with F-actin ring structures, though in significantly lesser quantity as compared to RANKL (*P* < 0.05) (Figure 4b). Whereas LTB4 activation formed more than 3-fold more giant TRAP^+^ multinuclear cells as compared to M-CSF alone (LTB4: 48 ± 10 cells, M-CSF: 15 ± 3 cells, *P* < 0.05), RANKL was able to form about 20-fold more osteoclast-like cells (234 ± 48 cells, *P* < 0.01). Combined treatment with RANKL and LTB4 significantly elevated the TRAP^+^ count to 530 ± 179 cells over LTB4 treatment alone (*P* < 0.01) (Figure 4b). Furthermore, these PBMCs treated with either M-CSF + RANKL or M-CSF + LTB4 for 14 days were equally capable of forming distinct F-actin ring structures as evidenced by phalloidin-FITC staining (Figure 4c).

## Discussion

**Figure 5:**
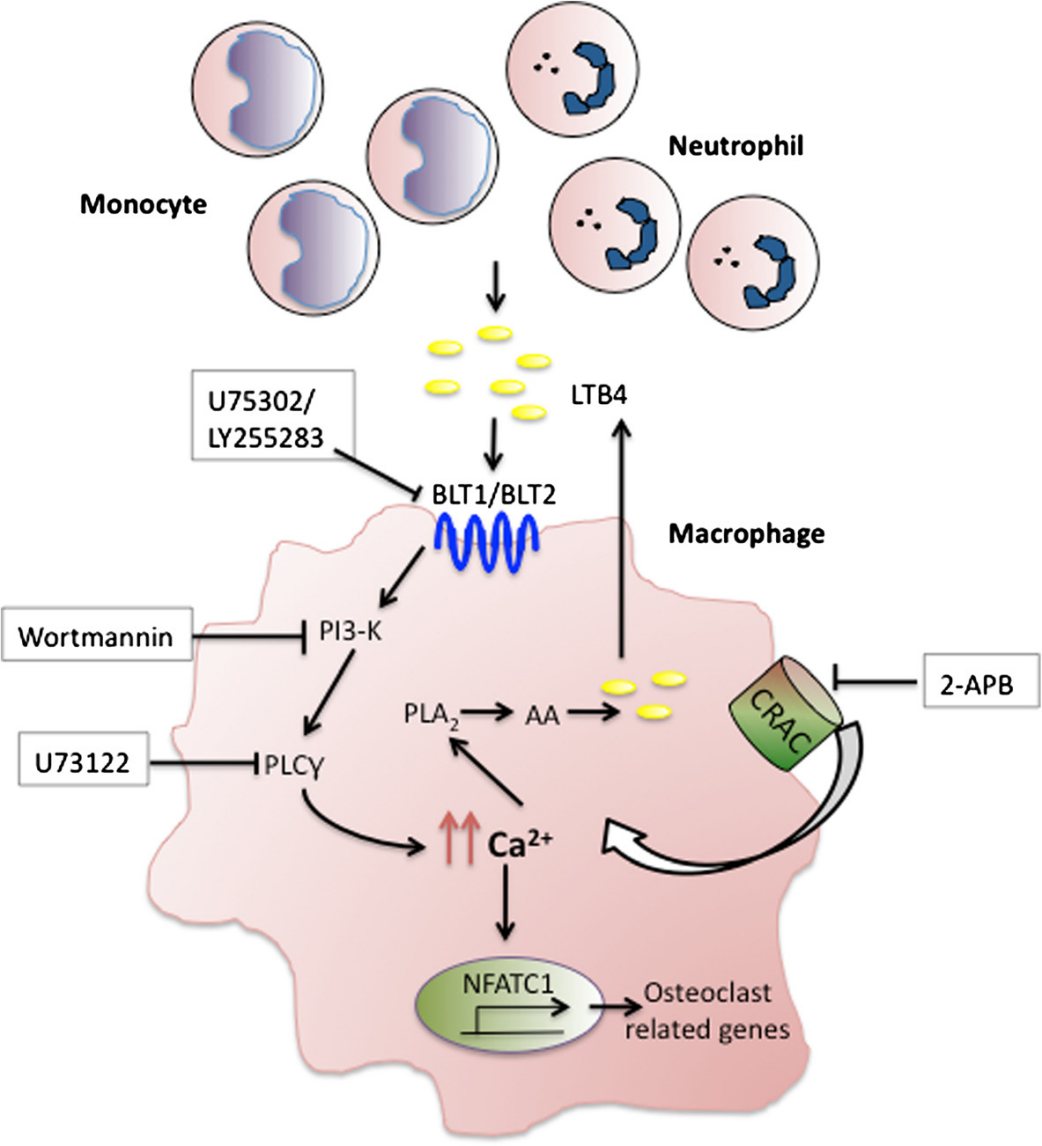
**Schematic representation of leukotriene B4 signaling events augmenting osteoclastogenesis.** Interleukin 23 (IL-23) induction of neutrophils and monocytes leads to the release of leukotriene B4 (LTB4), which associates with its G protein–coupled receptors (GPCRs) BLT1/BLT2 on macrophages to initiate calcium flux via cooperation between phospholipase C (PLC) and calcium release–activated channel (CRAC). Elevated intracellular calcium can then activate nuclear factor of activated T-cells, cytoplasmic 1 (NFATC1), and trigger osteoclastogenesis and also phosphorylate phospholipase A_2_ (PLA_2_) to further stimulate the production of LTB4 via an autocrine pathway. Thus, these pathways can lead to continuous production of LTB4, leading to enhanced osteoclastogenesis and exacerbation of the inflammatory milieu. To simplify the diagram, IL-23R pathway is not depicted in the schematic. AA, Arachidonic acid; 2-APB, 2-Aminoethoxydiphenyl borate; PI3-K, Phosphatidylinositol 3-kinase.

Alternate pathways for osteoclastogenesis have recently been a key focus for developing novel therapies for autoimmune arthritis. Although the leukotriene pathway has previously been shown to activate osteoclast formation, the precise mechanism of this differentiation and relationship with other critical inflammatory players, such as IL-23, has remained unexplored [31]. In this study, we demonstrate that IL-23 is an important activator of LTB4 production, which can significantly direct macrophages toward osteoclast differentiation. We highlight a novel pathway by which IL-23 can initiate LTB4 production from myeloid cells as well as drive their terminal differentiation to osteoclasts (Figure 5).

We have previously shown a distinct link between IL-23 and neutrophil activation, highlighting the innate immune system axis in rheumatoid arthritis [5]. Neutrophils are abundantly activated in an inflammatory response and play a key role in exacerbating inflammation in inflammatory arthritis [32,33]. Although they release several cytokines, such as IL-1β, IL-6 and TNF, their release of lipid inflammatory mediators, such as prostaglandins and leukotrienes, also contributes effectively to recruit neutrophils to inflamed joints [17]. Although this may be their primary and most well-characterized function, leukotrienes also act on effector cells via their BLT1/BLT2 receptors and activate other cell types [34,35]. Furthermore, leukotrienes may also be released from monocyte/macrophage populations, which, in the arthritis model, may lead to continuous autocrine production of LTB4 and enhanced osteoclastogenesis from macrophage precursors. Although both IL-23 and LTB4 are known separately for their inflammatory potential, this study demonstrates a novel finding of IL-23 stimulating LTB4 synthesis in myeloid cell populations present in our cultured human PBMCs. In physiologic conditions, this may function not only to recruit neutrophils to joint spaces and exacerbate the inflammatory conditions but also to act as a complementary secondary pathway for continuous osteoclast differentiation leading to bone loss.

Herein we demonstrate the presence of LTB4 receptors and LTB4 biosynthetic enzymes in macrophages as well as fully matured, giant multinuclear cells. We show that LTB4 autocrine activity also provides for continuous osteoclast differentiation via BLT1/BLT2 receptors on macrophages due to PLA_2_ activation. Furthermore, we also demonstrate that IL-23 phosphorylates PLA_2_ in macrophages to facilitate LTB4 production. IL-23 may trigger macrophages to release a variety of proinflammatory cytokines such as TNF and IL-1β, which can also activate PLA_2_ [36,37]. However, neither *in vivo* nor *in vitro* overexpression of IL-23 significantly altered soluble RANKL (sRANKL), TNF or IL-1β levels [5]. In keeping with our observations, other groups have also shown that IL-23 induced osteoclastogenesis in the absence of exogenous sRANKL in human PBMCs [38]. Similarly, in our *in vitro* system, IL-23 in the absence of exogenous sRANKL dose-dependently induced osteoclast formation, and enzyme-linked immunosorbent assay (ELISA) analysis of the conditioned medium did not detect sRANKL in the conditioned medium, confirming these findings. Although other groups have confirmed these findings, an upregulation of RANK mRNA expression following IL-23 stimulation of monocytes has been observed [39]. Therefore, it may be possible that sensitized myeloid cells respond to low levels of RANKL (undetectable by ELISA). Nevertheless, IL-23 induction of RANKL is not as important as the fact that IL-23 can induce RANKL-independent osteoclastogenesis via the regulation of an IL-17 and TNF mechanism [40].

Our data demonstrate a pathway where IL-23 can activate the phosphorylation of PLA_2_ via a PI3K-calcium flux–dependent pathway, thereby highlighting an important alternate mechanism by which LTB4 produced from macrophages induces osteoclastogenesis. This activation pathway, coupled with LTB4’s own autocrine ability, can lead to exacerbation of inflammatory conditions and bone loss in autoimmune arthritis.

IL-23 is capable of activating calcium transients in macrophages and these transients are critical for calcineurin dependent NFATC1 activation [41]. NFATC1 is a key transcriptional factor involved in osteoclastogenesis and is tightly regulated via calcineurin, a calcium dependent phosphatase responsible for activating NFATC1 and allowing its translocation to the nucleus [41]. We also show that LTB4 can activate significant calcium flux via its receptors and this activity is dependent on both internal and external sources of calcium as demonstrated by the use of inhibitors to phospholipase C and membrane bound CRAC channels [28]. Usage of the 2-APB inhibitor requires caution as it has been shown to block both store operated calcium entry (SOCE) as well as inositol triphosphate–gated channels within calcium stores [42]. However, at concentrations as high as 100 μM, which we used in the present study, 2-APB has been shown to effectively inhibit SOCE [43]. Recently, the store-operated calcium channel Orai1 and certain transient receptor potential channels have been shown to be important calcium channels involved in osteoclast activation; therefore, the regulation of calcium channels by inflammatory mediators may play a critical role in bone destruction [27,44,45]. Indeed, LTB4 was able to facilitate osteoclast development, as evidenced by the formation of multinucleated TRAP^+^ cells that were capable of F-actin ring formation. Moreover, LTB4-mediated calcium signaling was capable of activating the NFATC1 transcription factor and initiating the transcription of osteoclast-related genes such as *cathepsin K, MMP9, TRAP (ACP5)* and *β*_*3*_*integrin (ITGB3)*, which are all required for bone resorption. In our *in vitro* experiments, treatment with LTB4 produced significantly fewer osteoclasts and required a longer time as compared to RANKL stimulation. Activation with both RANKL and LTB4 achieved more TRAP^+^ multinucleated cells, as well as more cells fluxing calcium, in response to the dual stimulus. This implicates LTB4 as a definite proinflammatory, costimulatory signal in the development of arthritis in the presence of RANKL.

## Conclusions

Our study reveals novel links between IL-23 signaling and LTB4 activation that portrays the importance of the innate immune response in building an inflammatory milieu during the onset of autoimmune arthritis. IL-23 can facilitate the release of LTB4 from myeloid cells, which then can direct macrophages toward giant multinuclear osteoclasts independently of RANKL stimulation. Both LTB4 and IL-23 can activate PLA_2_ in macrophages, which leads to a continuous production of LTB4, thereby further heightening the inflammatory response. Together with RANKL, LTB4 acts as an important costimulatory signal and is a prominent target to develop effective therapies in inflammatory arthritis.

## Abbreviations

2-APB: 2-Aminoethoxydiphenyl borate
CRAC: Calcium release–activated channel
DAPI: 4′,6-diamidino-2-phenylindole
EIA: Enzyme-linked immunoassay
ELISA: Enzyme-linked immunosorbent assay
FITC: Fluorescein isothiocyanate
GPCR: G protein–coupled receptor
IL: Interleukin
5-LO: 5-Lipoxygenase
LTA4H: Leukotriene A4 hydrolase
LTB4: Leukotriene B4
LTC4S: Leukotriene C4 synthase
MAPK: Mitogen-activated protein kinase
M-CSF: Macrophage colony-stimulating factor
MFI: Mean fluorescence intensity
MMP9: Matrix metalloproteinase 9
NFATC1: Nuclear factor of activated T-cells, cytoplasmic 1
PBMC: Peripheral blood mononuclear cell
PBS: Phosphate-buffered saline
PFA: Paraformaldehyde
PI3K: Phosphatidylinositol 3-kinase
PLA_2_: Phospholipase A_2_
PLC: Phospholipase C
RANKL: Receptor activator of nuclear factor κB ligand
RT: Room temperature
SOCE: Store-operated calcium entry
TNF: Tumor necrosis factor
TRAP: Tartrate-resistant acid phosphatase
